# Increased colonic K^+^ excretion through inhibition of the H,K-ATPase type 2 helps reduce plasma K^+^ level in a murine model of nephronic reduction

**DOI:** 10.1038/s41598-021-81388-0

**Published:** 2021-01-19

**Authors:** Christine Walter, Chloé Rafael, Anthony Genna, Stéphanie Baron, Gilles Crambert

**Affiliations:** 1 Centre de Recherche des Cordeliers, INSERM, Sorbonne Université, Université de Paris, 75006 Paris, France; 2 CNRS ERL 8228 - Laboratoire de Physiologie Rénale et Tubulopathies, 75006 Paris, France; 3 Laboratoire de Physiologie, APHP, Hôpital Européen Georges Pompidou, 75015 Paris, France; 4 Centre de Recherche des Cordeliers - Laboratoire de Physiologie Rénale et Tubulopathies, 15 Rue de l’Ecole de Médecine, 75270 Paris Cedex, France; 5 Present Address: Anthony Genna - Bât. B23 Cancer-Tumours and Development Biology, Avenue de l’Hôpital 3, 4000 Liège, Belgium

**Keywords:** Nephrology, Chronic kidney disease, End-stage renal disease

## Abstract

Hyperkalemia is frequently observed in patients at the end-stage of chronic kidney disease (CKD), and has possible harmful consequences on cardiac function. Many strategies are currently used to manage hyperkalemia, one consisting of increasing fecal K^+^ excretion through the administration of cation-exchange resins. In this study, we explored another more specific method of increasing intestinal K^+^ secretion by inhibiting the H,K-ATPase type 2 (HKA2), which is the main colonic K^+^ reabsorptive pathway. We hypothetised that the absence of this pump could impede the increase of plasma K^+^ levels following nephronic reduction (N5/6) by favoring fecal K^+^ secretion. In N5/6 WT and HKA2KO mice under normal K^+^ intake, the plasma K^+^ level remained within the normal range, however, a load of K^+^ induced strong hyperkalemia in N5/6 WT mice (9.1 ± 0.5 mM), which was significantly less pronounced in N5/6 HKA2KO mice (7.9 ± 0.4 mM, *p* < 0.01). This was correlated to a higher capacity of HKA2KO mice to excrete K^+^ in their feces. The absence of HKA2 also increased fecal Na^+^ excretion by inhibiting its colonic ENaC-dependent absorption. We also showed that angiotensin-converting-enzyme inhibitor like enalapril, used to treat hypertension during CKD, induced a less severe hyperkalemia in N5/6 HKA2KO than in N5/6 WT mice. This study therefore provides the proof of concept that the targeted inhibition of HKA2 could be a specific therapeutic maneuver to reduce plasma K^+^ levels in CKD patients.

## Introduction

Hyperkalemia is an electrolytic disorder that may severely affect cardiovascular functions (for review see^1^). According to multiple studies^2–6^ the incidence of hyperkalemia is increased in patients suffering end-stage chronic kidney disease (CKD) with glomerular filtration rate below 30 ml/min/1.73m^2^, reaching 40% in the presence of comorbidities such as diabetes. In CKD patients, plasma K^+^ level is also impacted by anti-hypertensive treatments that affect the renin–angiotensin–aldosterone system (RAAS)^2–6^ such as angiotensin-converting-enzyme (ACE) inhibitors (enalapril…), angiotensin II receptor blockers (losartan…) or mineralocorticoid receptor antagonist (epleronone…). The development of hyperkalemia can be explained by the fact that some CKD patients maintain their K^+^ balance (equilibrium between K^+^ input and output) to the detriment of the plasma K^+^ level. Hyperkalemia is correlated with a higher mortality rate due to cardiovascular events^4,7–9^. To reduce plasma K^+^ level in CKD patients, the most frequent strategies consist in buffering the dietary K^+^ by ingestion of cationic resins (sodium polystyrene sulfonate or others) and/or by the discontinuation/suppression of RAAS inhibitor medications, which may result in negative consequences on the progression of the disease.

Maintaining the K^+^ balance requires coordinated regulation of the molecular mechanisms that store and release K^+^ from internal stores (internal balance) and those that retain and excrete K^+^ (external balance) (for recent reviews see^10,11^). In a normal context, the colon contributes to K^+^ homeostasis through its ability to either reabsorb or secrete K^+^, however its involvement is minor compared to the kidneys since, under a normal K^+^ diet, less than 10% of the K^+^ intake is excreted in the feces. Conversely, during end-stage CKD, the colonic contribution to K^+^ homeostasis becomes more crucial^12,13^.

In the colon, the transport of K^+^ includes both secretion and reabsorption pathways^14,15^. The secretion primarily depends on Na,K-ATPase and Na–K–2Cl-cotransporter (NKCC1) at the basal side of the cells and on Ca^2+^-activated or cAMP-activated K^+^ channels at the apical membrane of the cells. The secretion is in part dependent on aldosterone that stimulates the expression of the Na,K-ATPase and K^+^ channels^16–18^. In the colon, aldosterone also stimulates the expression of the β and γ subunits of the epithelial Na^+^ channel (ENaC), but not the α subunit^19–22^. Noteworthy, contrary to its activity in the kidney, ENaC is not a strong determinant of colonic K^+^ secretion since this process is mainly insensitive to amiloride^23,24^ and the fecal excretion of K^+^ is not altered in ENaC α subunit KO mice^25^.

The reabsorption of K^+^ is mediated by the H,K-ATPase type 2 (HKA2), an electroneutral transporter^26^ consisting of two subunits, a catalytic α subunit (encoded by the *Atp12a* gene) that is associated with a β subunit (encoded by the *Atp1b1* gene, in the colon^27^). In heterologous systems, however, different chaperon-like β subunits may associate with the α subunit to form an active HKA2^28,29^. This transporter exhibits pharmacological and transport features common to two closely related P-type ATPases, the Na,K-ATPase and the H,K-ATPase type 1 (for review see^30^). For instance, HKA2 may transport Na^+^ instead of H^+^ in the kidney^28,31,32^, is sensitive to ouabain^33–35^ like the Na,K-ATPase, and contributes to the renal secretion of salt^36,37^. It also transports H^+^ and is sensitive to Schering 28080^38^ like the H,K-ATPase type 1. HKA2 participates at the K^+^ and Na^+^ balance during the circadian cycle^39^ and in different conditions like K^+^ depletion^40,41^ and gestation^42,43^. Because of the strong expression of HKA2 in the distal colon, as well as the remarkable fecal K^+^ loss phenotype of HKA2-deficient mice^44^, we drew the hypothesis that the absence of the HKA2 could be beneficial in the context of end-stage renal disease by facilitating the elimination of K^+^ in the feces and limiting the development of hyperkalemia.

## Results

### Colonic expression of the HKA2 is not altered by the nephronic reduction

**Figure 1:**
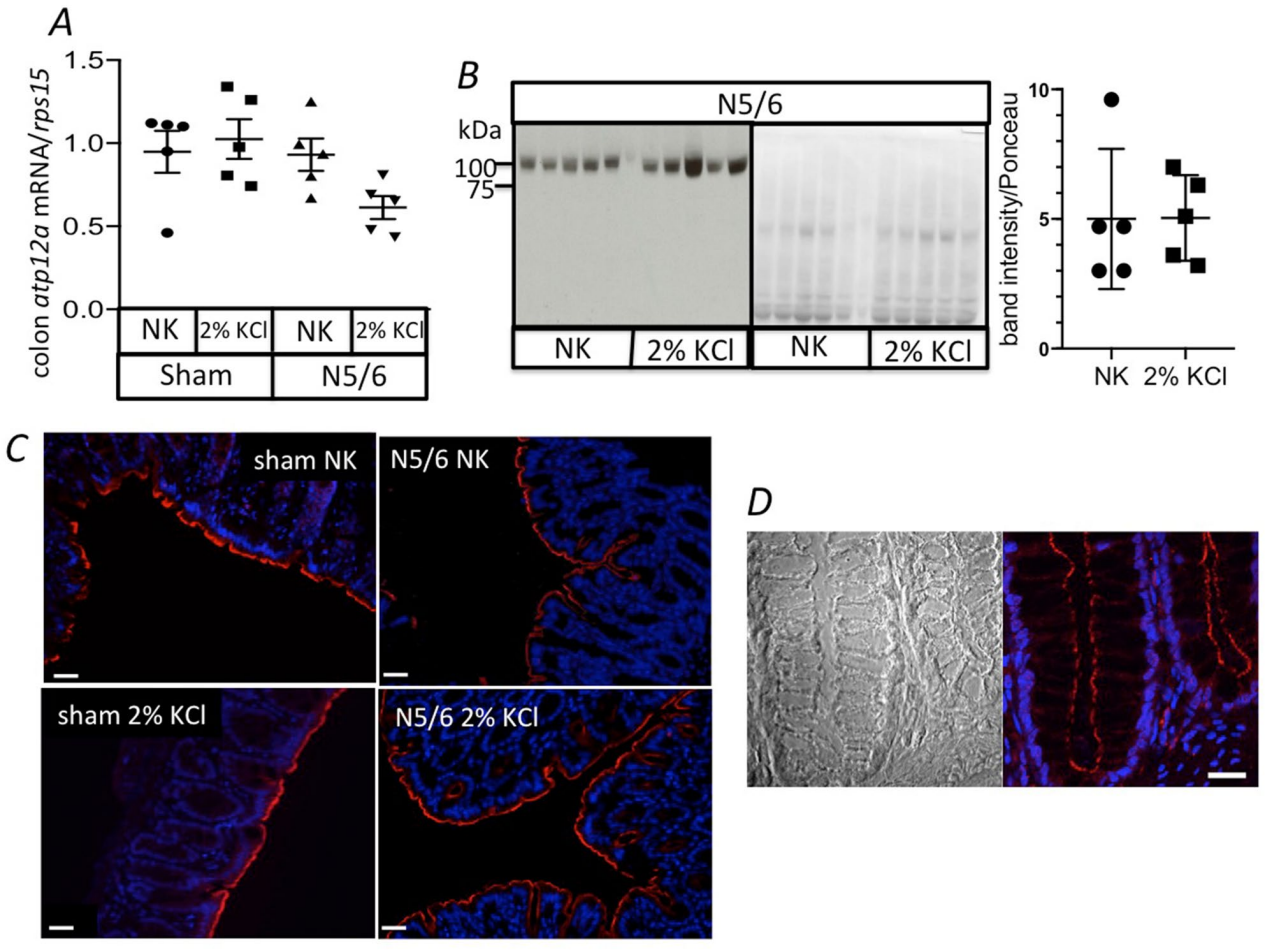
Expression of HKA2 in colon is not influenced by nephronic reduction (N5/6) and K^+^ load. (**A**) mRNA expression of the *Atp12A* gene encoding for the catalytic subunit of the HKA2 in colon of sham or N5/6 WT mice under normal diet (NK) or after a 24 h load of KCl (2% in drinking water) normalized by the housekeeper gene *Rps15*. Results are shown as the mean ± s.e.m (n = 5). (**B**) Western blot detection of the HKA2 α subunit (left panel) in N5/6 mice under NK or after a 24 h load of KCl (2% KCl). The signal is normalized with the Ponceau S Red labeling (right panel). Results are shown as the mean ± s.e.m (n = 5). (**C**) Labelling of the HKA2 α subunit (red) and of nucleus (DAPI, blue) on colon slice from sham and N5/6 mice under NK or after a 24 h load of KCl (2% KCl). For all slides, the pictures were taken with the same parameters of exposition and magnification (×20). Scale bars: 20 µm. D/ Brightfield and labelling of the HKA2 α subunit (red) and of nucleus (DAPI, blue) on normal colon slice from human subjects.

The presence of HKA2 in colon is well-established (for review see^30^) but its level of expression or its localization could be altered by different physiological or pathophysiological contexts. Since our hypothesis is based on the possibility that a lack of the HKA2 in the colon might improve the excretion of K^+^ in nephrectomized mice, we first investigated that its expression is not altered by 5/6 nephronic reduction (N5/6). As shown in Fig. 1A–C, the nephronic reduction and the increased intake of K^+^ did not modify the mRNA expression of the *Atp12a* gene (encoding the catalytic subunit of the HKA2), or the HKA2 protein level or localization in the colon. The presence of HKA2 in the human colon has not been extensively investigated, but its mRNA expression has been reported^45,46^. To support the relevance of our study, we showed in Fig. 1D that HKA2 localized to the apical side of colonocytes in the human colon. Moreover, we showed that nephronic reduction induced a similar raise of plasma creatinine (Table 1) in WT and HKA2KO mice.

**Table 1:** Physiological parameters of WT or HKA2KO mice, sham or nephrectomised (N5/6) under normal (NK) or high potassium (2% KCl) diet for 24 h.

	NK				2% KCl			
	WT		HKA2KO		WT		HKA2KO	
	Sham	N5/6	Sham	N5/6	Sham	N5/6	Sham	N5/6
Weight (g)	25.3 ± 0.9	22.8 ± 0.6	26.2 ± 1.0	24.6 ± 0.5	25 ± 0.9	21.6 ± 0.6	25.5 ± 0.9	23.8 ± 0.4
Food (g)	3.2 ± 0.4	3.7 ± 0.1	4.0 ± 0.2	3.9 ± 0.2	3.8 ± 0.2	2.5 ± 0.1**	4.2 ± 0.3	2.3 ± 0.1**
Water (ml)	3.8 ± 0.2	6.8 ± 0.6*	3.8 ± 0.3	7.9 ± 0.5**	5.6 ± 1.3	6.2 ± 0.6	5.2 ± 0.3	6,7 ± 0.8
Urine vol. (ml)	1.2 ± 0.2	2.6 ± 0.4*	1.5 ± 0.2	3.7 ± 0.4**^##^	1.8 ± 0.2	4.2 ± 0.3**	2.5 ± 0.3	4.4 ± 0.4**
Feces (g/day)	1.3 ± 0.1	1.4 ± 0.1	1.7 ± 0.2#	1.4 ± 0.1	1.0 ± 0.1	0.8 ± 0.1	1.5 ± 0.1	0.8 ± 0.1
Plasma creat. (mM)	11.4 ± 0.6	21.1 ± 1.0**	8.5 ± 0.7	25.3 ± 2.2**	N.D	N.D	N.D	N.D

Results are shown as mean ± s.e.m. Two-way ANOVA test followed by a Sidak’s multiple comparisons test, (sham vs N5/6 **p < 0.01; *p < 0.05—WT vs. HKA2KO ^##^*p* < 0.01; ^#^*p* < 0.05).

### The absence of HKA2 increases fecal K^+^ excretion and limits the development of hyperkalemia after an acute increase of K^+^ intake

**Figure 2:**
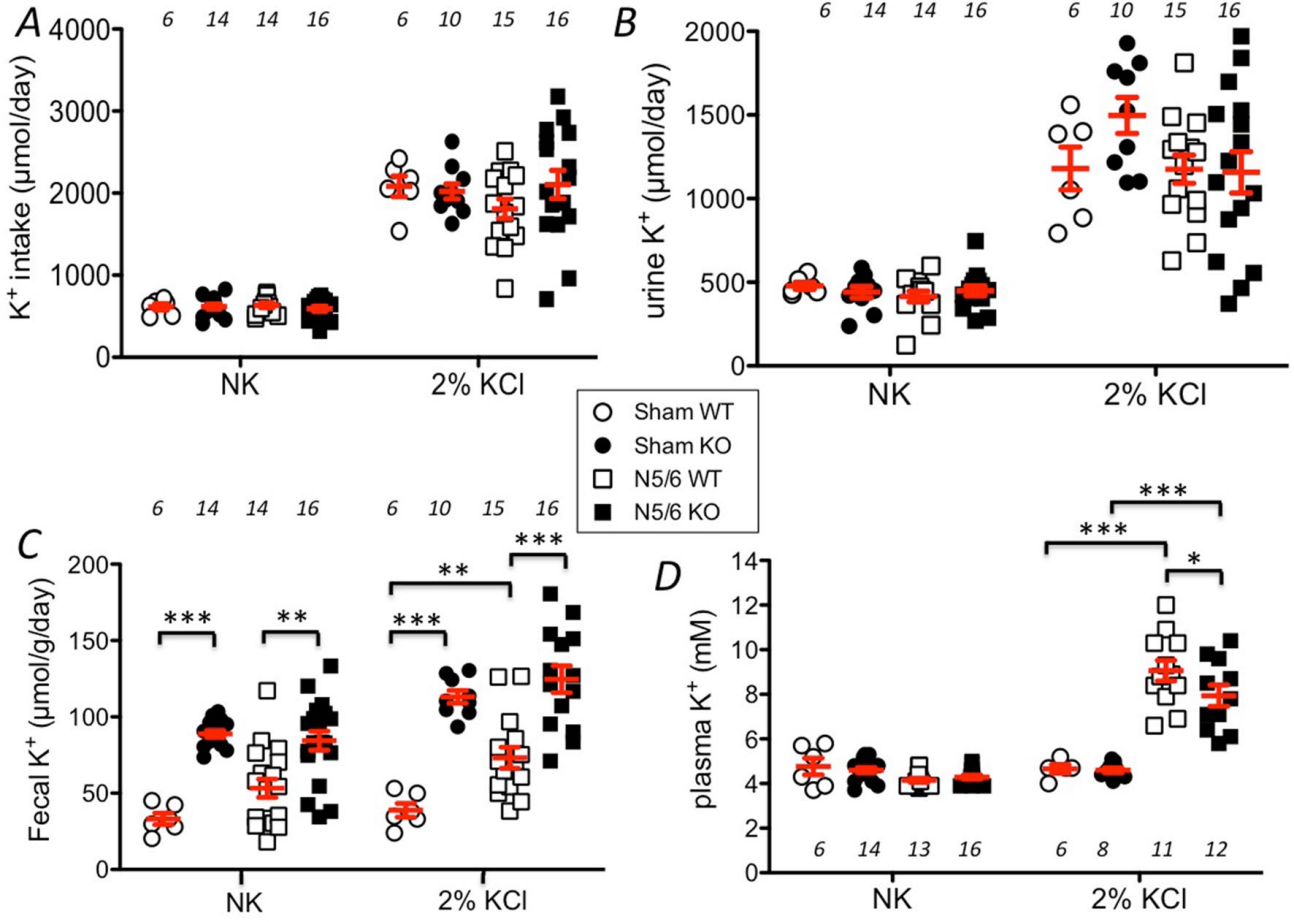
Parameters of the K^+^ balance. The absence of HKA2 limits the increase of plasma K^+^ level. (**A**) Daily K^+^ intake in sham WT (white circles), sham HKA2KO (black circles), N5/6 WT (white squares) and N5/6 HKA2KO (black squares) mice. Results are shown as the mean ± s.e.m. Numbers in italic = n of mice. (**B**) Daily urine K^+^ excretion in sham WT (white circles), sham HKA2KO (black circles), N5/6 WT (white squares) and N5/6 HKA2KO (black squares) mice. Results are shown as the mean ± s.e.m. Numbers in italic = n of mice. (**C**) Daily fecal K^+^ excretion in sham WT (white circles), sham HKA2KO (black circles), N5/6 WT (white squares) and N5/6 HKA2KO (black squares) mice. Results are shown as the mean ± s.e.m. Numbers in italic = n of mice. Two-way ANOVA test followed by a Sidak’s multiple comparisons test, (**p < 0.01; *p < 0.05). D/ Plasma K^+^ value in sham WT (white circles), sham HKA2KO (black circles), N5/6 WT (white squares) and N5/6 HKA2KO (black squares) mice. Results are shown as the mean ± s.e.m. Numbers in italic = n of mice. Two-way ANOVA test followed by a Sidak’s multiple comparisons test, (**p < 0.01; *p < 0.05).

**Figure 3:**
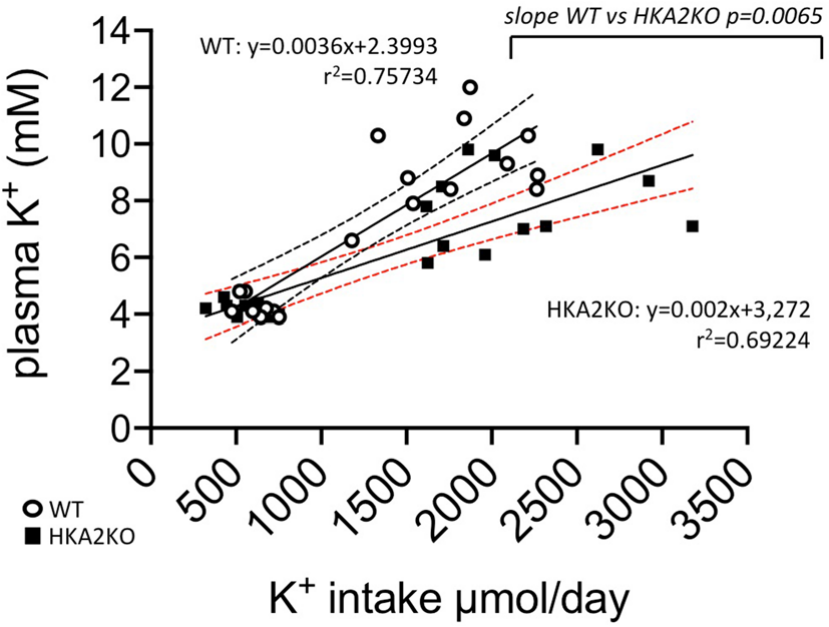
HKA2KO mice exhibit a lower sensitivity to K^+^ intake than WT mice. Relationship between daily K^+^ intake and plasma K^+^ value in N5/6 WT (white circles) and in HKA2KO mice (black squares). Results are shown as individual points fitted with a least squares regression method (straight lines) in a 95% confidence intervals (dotted lines).

The absence of HKA2 has been shown to affect the K^+^ balance^39,43,44^. To test whether the absence of HKA2 may influence the plasma K^+^ level in N5/6 mice, we compared sham WT and HKA2KO with N5/6 WT and HKA2KO mice under normal diet (NK) or after a 24 h K^+^ loading with 2% KCl in the drinking water. The general parameters are displayed in Table 1 and showed no statistical difference in the weight of mice between groups. Regarding food intake, it decreased by 34% in N5/6 WT mice under 2% KCl compared to the sham WT in the same condition, and by 45% in N5/6 HKA2KO mice under 2% KCl compared to the sham HKA2KO in the same condition. This effect is likely due to the increased level of plasma K^+^ value observed in these conditions (see below), an effect that has previously been observed in another context^47^. The daily urine volume was roughly doubled by nephrectomy in both genotypes and both potassium conditions. As shown in Fig. 2A, the total K^+^ intake (calculated from food and drinking water consumption) was 3 to fourfold increased following the addition of KCl to the drinking water (*p* < 0.01), and was similar in all groups under normal K^+^ diet (around 600 µmol/day) or in K^+^-loaded groups (around 2200 µmol/day). Urine K^+^ excretion (Fig. 2B) was almost 3-times higher in K^+^ loaded groups of mice than in mice under normal K^+^ diet (*p* < 0.01) but was not significantly modified by the nephrectomy and/or the absence of the HKA2. As expected, under normal K^+^ diet, the fecal K^+^ excretion (Fig. 2C) was 2–3 times higher in sham HKA2KO mice than in sham WT mice (88.8 ± 2.2 vs. 33.1 ± 3.8 µmol/g/day, respectively). This difference among genotypes was also observed in N5/6 mice but nephronic reduction itself did not significantly modify the fecal K^+^ excretion of WT or HKA2KO mice. After the 24 h K^+^-load, fecal K^+^ excretion remained almost 4-times higher in sham HKA2KO mice than in sham WT mice (113.1 ± 4.1 vs. 38.9 ± 4.5 µmol/g/day, respectively). The nephrectomy increased the fecal K^+^ excretion in WT by 80% (from 38.9 ± 4.5 µmol/g/day in 2% KCl, sham WT to 73.2 ± 6.9 µmol/g/day N5/6 WT, *p* < 0.01) but remained significantly lower than in N5/6 HKA2KO (124.6 ± 8.8 µmol/g/day). Under the normal K^+^ diet, the plasma K^+^ level (Fig. 2D) of sham and N5/6 mice, whatever their genotype, is similar and in the normal range (around 4.2 mM for the four groups). After an acute load of K^+^, the plasma K^+^ level remained unchanged in sham mice. Conversely, this treatment induced a strong increase of plasma K^+^ value in N5/6 WT mice (9.1 ± 0.5 mM), which was significantly less pronounced in N5/6 HKA2KO mice (7.9 ± 0.4 mM, *p* < 0.01). To better characterize the sensitivity of nephrectomized mice to K^+^, we plotted the relationship between plasma K^+^ level vs total K^+^ intake (Fig. 3). The N5/6 WT mice displayed an almost 2 times higher sensitivity to K^+^ intake than N5/6 HKA2KO mice (+ 0.36 mM/100 µmol of increase K^+^ intake and + 0.2 mM/100 µmol of increase K^+^ intake in WT and HKA2KO mice, respectively, *p* < 0.01).

### The absence of the HKA2 limits the colonic Na^+^ absorption by modulating ENaC expression

**Figure 4:**
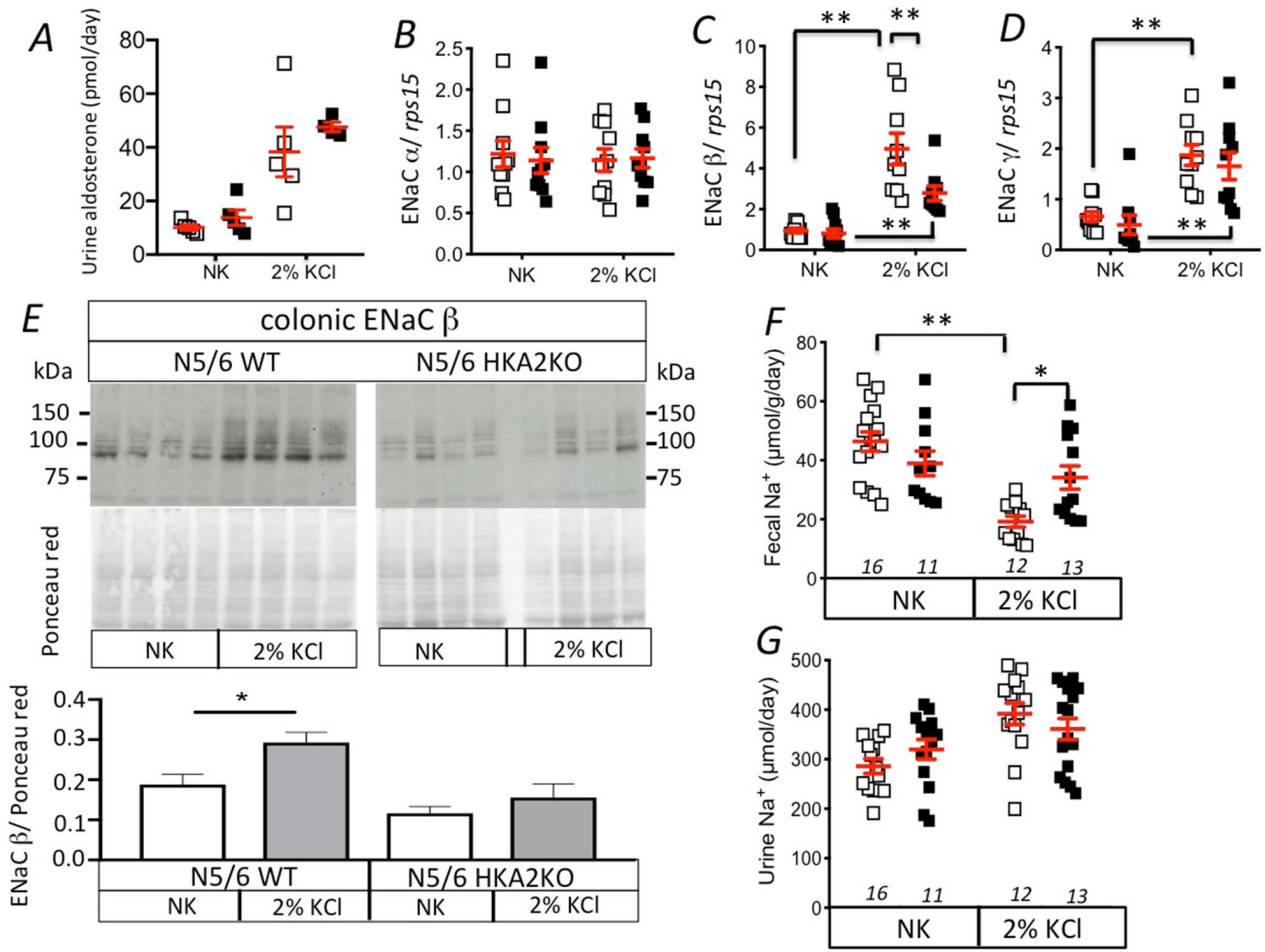
Colonic ENaC expression and fecal Na^+^ excretion are also altered in N5/6 HKA2KO mice. (**A**) Daily urine aldosterone excretion in N5/6 WT (white squares) and N5/6 HKA2KO (black squares) mice under normal (NK) or high K^+^ intake (2% KCl). Results are shown as the mean ± s.e.m, n = 5. (**B**–**D**) mRNA expression of ENaC α, β and γ subunits in N5/6 WT (white squares, n = 10) and N5/6 HKA2KO (black squares, n = 10) mice under normal (NK) or high K^+^ intake (2% KCl). Results are shown as the mean ± s.e.m. (**E**) *higher panel*, Immunoblot of the ENaC β subunit in N5/6 WT (white squares, n = 4) and N5/6 HKA2KO (black squares, n = 4) mice under normal (NK) or high K^+^ intake (2% KCl). *Middle panel*, Ponceau red labeling of the membrane. *Lower panel*, quantification of the band intensity normalized with the Ponceau red labeling. (**F**) Fecal excretion of Na^+^ in N5/6 mice under normal (NK) or high K^+^ intake (2% KCl). (**G**) Urine excretion of Na^+^ in N5/6 mice under normal (NK) or high K^+^ intake (2% KCl). Results are shown as the mean ± s.e.m. Numbers in italic = n of mice. Two-way ANOVA test followed by a Sidak’s multiple comparisons test, (**p < 0.01; *p < 0.05).

Aldosterone is a steroid hormone that is involved in both K^+^ and Na^+^ balances^48^. The decrease of the K^+^ sensitivity in N5/6 HKA2KO mice may result in changes of aldosterone level and Na^+^ homeostasis. As shown in Fig. 4A, the aldosterone levels of N5/6 WT and HKA2KO mice are similar in normal conditions and are similarly increased (4-times) by a K^+^ load. Despite this similar level in aldosterone, we observed differences in the colonic expression of ENaC subunits between WT and HKA2KO mice. As shown in Fig. 4B–D, the mRNA expression of the ENaC α subunit was similar in N5/6 WT and HKA2KO mice whatever the K^+^ intake and that of the ENaC γ subunit was similarly increased by K^+^ loading in N5/6 WT and HKA2KO mice. Regarding the ENaC β subunit, its mRNA expression in the colon of N5/6 WT and HKA2KO mice was differentially stimulated by K^+^ intake. Indeed, it was significantly 2 times lower in HKA2KO mice than in WT mice (*p* < 0.01). At the protein level, we confirmed that the level of the β subunit of ENaC was upregulated in N5/6 WT mice after K^+^ loading, which was not the case in N5/6 HKA2KO mice (Fig. 4E).

As shown in Fig. 4F, in nephrectomized mice, the fecal Na^+^ excretion is similar between WT and HKA2KO mice under the normal K^+^ diet. In N5/6 WT mice, K^+^ loading induced a 2.5-fold decrease in fecal Na^+^ excretion whereas N5/6 HKA2KO mice did not display any modification of their fecal Na^+^ excretion. The absence of HKA2, therefore, impedes colonic Na^+^ retention in response to nephrectomy and K^+^ loading.

### The absence of the HKA2 limits the development of hyperkalemia induced by pharmacological treatment

**Figure 5:**
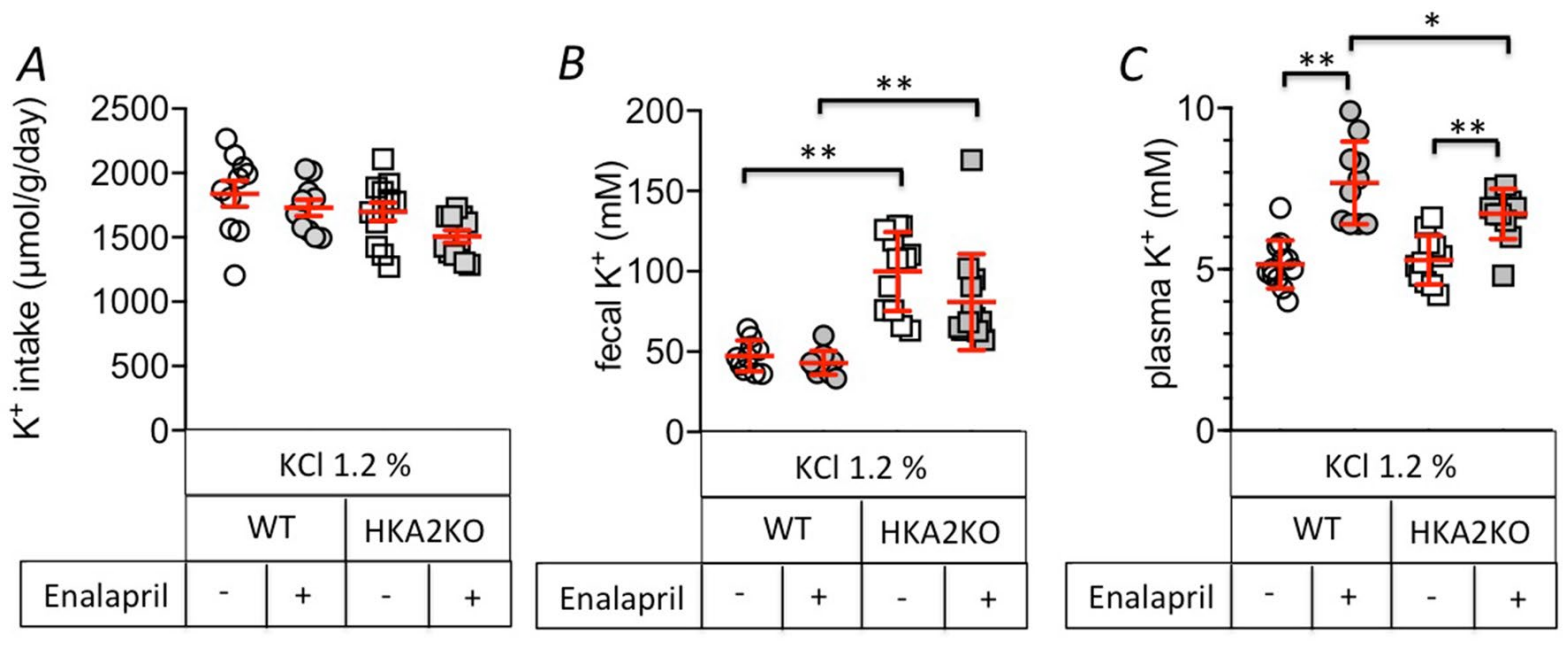
Enalapril-induced hyperkalemia is lowered in N5/6 HKA2KO mice. (**A**) Daily K^+^ intake in N5/6 WT (circles) or HKA2KO (squares) mice after 24 h-K^+^ loading treated (grey symbols) or not (white symbols) with enalapril (0.12 mg/ml) for a week. (**B**) Fecal K^+^ excretion in N5/6 WT (circles) or HKA2KO (squares) mice after 24 h-K^+^ loading, treated (grey symbols) or not (white symbols) with enalapril (0.12 mg/ml) for a week. (**C**) Plasma K^+^ values in N5/6 WT (circles) or HKA2KO (squares) mice after 24 h-K^+^ loading, treated (grey symbols) or not (white symbols) with enalapril (0.12 mg/ml) for a week. Results are shown as the mean ± s.e.m (n = 10). Two-way ANOVA test followed by a Sidak’s multiple comparisons test, (**p < 0.01; *p < 0.05).

As mentioned above, the molecules used for treating hypertension, such as inhibitors of the angiotensin-converting enzyme (ACE), are capable to induce hyperkalemia^49^. Therefore, we treated N5/6 mice for a week with enalapril or not, and then, all groups (treated or not) were loaded with 1.2% KCl in the drinking water. The enalapril treatment was similar in N5/6 WT and HKA2KO mice (0.82 and 0.76 mg/day, corresponding to 34 and 33 mg/kg/day, respectively). As shown in Fig. 5A, all groups ingested a similar amount of K^+^ (around 1700 µmol/day, 30% less than in Fig. 2A). Per se, this amount did not induce hyperkalemia in either N5/6 WT or N5/6 HKA2KO mice (Fig. 5B), although the plasma K^+^ value was approximately 1 mM higher than that in N5/6 mice under normal K^+^ diet (Fig. 2D). Fecal K^+^ excretion was not modified by the treatment with enalapril and remained higher in HKA2KO than in WT mice. The treatment with enalapril increased the plasma K^+^ level (Fig. 5C) to 7.7 ± 0.4 mM in N5/6 WT mice, which was significantly higher than that in N5/6 HKA2KO mice (6.7 ± 0.2 mM, *p* < 0.05). The absence of HKA2 is, therefore, also efficient to impede the increase of plasma K^+^ level induced by an anti-hypertensive treatment.

## Discussion

Reduced renal function is associated with many electrolytic disturbances^50^. In this context, the prevalence of hyperkalemia is very important and is one of the main reasons for discontinuation or dose reduction of RAAS inhibitors^9^. The plasma K^+^ level is not the only factor that determines when hyperkalemia represents a risk^1^, its occurrence alongside acidosis, diabetes, low plasma Mg^2+^ and/or Ca^2+^ can potentiate the electrophysiological disturbances of cardiomyocytes, leading to arrhythmia and sudden death^9^. Therefore, reducing plasma K^+^ levels in patients with CKD can protect them from adverse cardiac effects. One of the most frequently prescribed strategies for reducing plasma K^+^ levels is the buffering of dietary K^+^ by cationic resins, to prevent its intestinal K^+^ absorption. For 60 years, the cationic resin used was a sodium polystyrene sulfonate, exchanging Na^+^ for K^+^^51^. However, the side effects and the poor compliance of patients to absorb this resin has led to the development of novel resins (Patiromer) or cation trapping agents (ZS-9) with less adverse effects and higher capacity of K^+^ binding (for review comparing SPS, ZS-9 and Patiromer, see^52^). However, none of the available resins are selective, and therefore can also deplete other cations than K^+^, and require the patients to take a large amount of the medication daily (few tens g/day).

Due to the adverse side effects and lack of selectivity of the available cationic resins, the search for a more specific mechanism of improving fecal excretion of K^+^ is relevant. In this study, we showed that inhibiting HKA2 decreased the plasma K^+^ level by around 1 mM in a mouse model of CKD in the context of hyperkalemia^53^ (Figs. 2D and 5C). This correlates to the increased fecal excretion of K^+^ observed in HKA2KO mice (Figs. 2C and 5B), which is not compensated for by the kidney, since the urine K^+^ excretion remains similar between WT and KO mice independent of K^+^ intake. The K^+^ depleted state of HKA2KO mice therefore appears to be an advantage in the context of CKD to help excrete K^+^. The decrease of plasma K^+^ value we have observed in this study is very similar to that obtained by the use of Patiromer resin in a rat model pharmacologically treated to be hyperkalemic^54^.

Interestingly, the elevation of aldosterone in response to hyperkalemia in N5/6 WT mice correlates with a lower fecal excretion of Na^+^ that could be attributed to the strong expression of the β and γ ENaC subunits and the stimulation of ENaC-mediated Na^+^ absorption (Fig. 4). In the HKA2KO mice, this decrease of fecal Na^+^ is not observed and could be attributed to a less substantial stimulation of the β ENaC subunit (Fig. 4). Moreover, in the context of hyperaldosteronism, it has also been proposed that the Na^+^ absorption in the colon depends on the recycling of K^+^ at the apical side through HKA2^55^. Therefore,the lack of HKA2 not only favors the fecal K^+^ excretion but also impedes the colonic Na^+^ retention. This last effect is also of particular interest in the context of CKD since salt retention and increased blood pressure are common and unfavorable features of the CKD.

Is the strategy of targeting HKA2 relevant in humans? The presence of HKA2 in the human colon was established 20 years ago^46^ and we showed that it is localized, as in mice, at the apical side of the colonocytes at the surface of the human colonic epithelium (Fig. 1D). The pharmacology of HKA2 is complicated and has not been fully investigated (for review see^30^). However, some data exist in the literature suggesting that HKA2 could be inhibited by the “proton-pump inhibitors” (PPIs) of the omeprazole family^56–59^. These compounds are well-known to inhibit the H,K-ATPase type 1, which is structurally and functionally closely related to HKA2. PPIs do not have a good reputation in CKD since their chronic use is correlated to a deterioration in renal function^60^. However, interestingly, the use of omeprazole has been found to be associated with a low plasma K^+^ level in CKD patients under peritoneal dialysis^61^, which may be explained through an effect on HKA2, leading to the inhibition of K^+^ retention. Recently, large screening of compounds with specific activity to block colonic K^+^ absorption as a potential treatment for hyperkalemia revealed a molecule with a proscillaridin A-like structure^62^. Interestingly, this family of compounds belongs to the cardiac glycosides family that inhibit the Na,K-ATPase but also has the potential to block HKA2. The development of compounds that specifically inhibit K^+^ reabsorption in the colon by interfering with HKA2 seems therefore a promising alternative to the use of resins. Ideally, since HKA2 is expressed in different tissues and organs and have been shown to participate at different physiological functions^43,63^, its inhibitor should be in a galenic form that impedes its intestinal absorption. It would therefore only target the colonic HKA2, which should avoid possible negative side effects. In CKD patients, the development of hyperkalemia is not only related to daily K^+^ intake but also to anti-hypertensive treatments, stage of the disease and yet unknown intrinsic factors, therefore, similarly to the use of cationic resins, we think that HKA2 inhibition should be initiated in face of an increase of plasma K^+^ level.

In conclusion, we showed that the inactivation of the HKA2 in the colon, inducing a fecal loss of K^+^, may help reducing plasma K^+^ level in front of dietary K^+^ loading or pharmaceutical hyperkalemic treatments. These data suggest that this ion pump could be an interesting target to help reduce the plasma K^+^ level in CKD patients.

## Material and methods

### Animals

Experiments were performed on C57BL/6J wild-type (from the Janvier Labs company, France) and mice with a HKA2 α subunit gene knock out^44^. The colony of HKA2KO mice is maintained by mating knock-out male and female mice. To avoid genetic deviation, every 5 generations, a backcross is performed with wild-type C57BL/6J males mice from the Janvier Labs. As recently mentioned^42^, all animals were kept at CEF (Centre d’Explorations Fonctionnelles of the Cordeliers Research Center, Agreement no. B75-06-12). All experiments were conducted in accordance with the institutional guidelines and the recommendations for the care and use of laboratory animals put forward by the Directive 2010/63/EU revising Directive 86/609/EEC on the protection of animals used for scientific purposes (project has been approved by the ethics committee “Charles Darwin” of Sorbonne Université—Project Authorization number 8242). The study was carried out in compliance with the ARRIVE guidelines. The nephronic reduction (N5/6) was performed on anesthetized (ketamine/xylazine 100 and 10 mg/kg, respectively) male mice. During the first surgery the two poles of the left kidney were removed (roughly 2/3 of the kidney). After a week of rest a second surgery was performed to entirely remove the right kidney. Buprenorphine (0.5 mg/kg) was given before, during and 24 h after the surgery to prevent pain. Sham animals underwent similar procedures except that their kidney remained intact. At this stage the influence of sex has not been investigated, we therefore only conducted the analysis with male mice.

### Physiological measurements

To record physiological parameters, the mice (sham or N5/6) were placed in metabolic cages (Techniplast, France) and were fed a standard laboratory diet for 7–10 days following the last surgery (0.3% Na^+^ and 0.6% K^+^; Safe France for more information regarding the detailed composition see http://www.safe-diets.com/wp-content/uploads/2018/01/DS-SAFE-A04.pdf). The food therefore contained 154.3 µmol/g of K^+^. K^+^ load was provided by adding 2% KCl to the drinking water for a period of 24 h. This corresponded to 268 µmol K^+^/ml. This method of administration was selected instead of adding K^+^ to the food because we anticipated a rise in plasma K^+^ levels, which is known to induce a loss of appetite and a decrease in food intake^47^. To test the effects of enalapril, N5/6 mice were treated with or without 0.12 mg/ml of enalapril (dissolved in the drinking water) for a week. This concentration was chosen according to Wang et al.^64^ and calculated to reach 30 mg/kg/day. On the 7th day of the enalapril treatment, all groups of mice were placed into metabolic cages and were given 1.2% KCl in their drinking water. We chose a lower K^+^ load for this experiment (1.2% instead of 2%) because we anticipated that the concomitant effects of nephrectomy, K^+^ load and enalapril treatment could result in excessive hyperkalemia. After a 24 h-period collection, urinary K^+^ concentration was determined by flame photometry (IL943, Instruments laboratory, France) and plasma parameters were measured by retro-orbital puncture on the anesthetized animal with an ABL77 pH/blood-gas analyzer (Radiometer, Lyon, France). Noteworthy, few measurements of plasma parameters failed due to either technical problems with the apparatus or difficulties in the blood collection. This is why the number of mice presented in Fig. 2D is slightly different for some groups compared to Fig. 2A–C. Stool was collected, brushed (to eliminate food contaminant), homogenized in distilled water (100 mg/300 µl) and centrifuged for 15 min at 10,000×*g*. The supernatant was collected, the pellet was homogenized again (300 µl distilled water) and treated in the same condition two times. Potassium contents were then measured on the three collected supernatants per sample by using a flame photometer (IL 943, Instruments Laboratory). Urine aldosterone concentration was determined with a chemiluminescent immunoassay (Diasorin, Salluggia, Italy) after 24 h pH 1 acid hydrolysis.

### Quantitative PCR

As previously mentioned^42^ RNA was extracted from whole tissue using the TRI reagent (Invitrogen, Villebon sur Yvette, France) following the manufacturer’s instructions. One µg of total RNA was then reverse-transcribed using the first strand cDNA synthesis kit for RT-PCR (Roche Diagnostics, France) according to the manufacturer’s instructions. Real-time PCR was performed on a LightCycler (Roche Diagnostics, France). No signal was detected in samples that did not undergo reverse transcription or in blank runs without cDNA. In each run, a standard curve was obtained using serial dilution of stock cDNA prepared from mouse kidney total RNA. The expression of the *Rps15* gene was used to normalize the results (mean threshold cycle for Rps15 = 23 ± 0.1). Specific primers for ENaC α (up CCAAACGAACCGAACAC; down TGTCAGACTTACTCTAGCC), ENaC β GGTCCTTATTGATGAGCG; down AGGCGTGAAGTTCCGA), ENaC γ (up TCGGTCGTCTGTGTCA; down GCAGATCATCGTCCGTAT), ATP12a (up TTGGAAACTAAGAACATAGGCTTCTATT; down AATGGCTATGGGTGTCTTCTCA) and rps15 (up TTTCCGAGTAACCGCC; down GCAGTGAGTGTTGCTT) transcripts were chosen using the LC Probe design 2.0 software.

### Membrane protein extraction and Western Blot analysis

As previously described^42^, the distal colons were homogenized in a lysis buffer (250 mM sucrose, 100 mM Tris-Hepes, pH 7.4 and protease and phosphate inhibitor cocktails (Complete, Roche Diagnostics)). After removal of aggregates and nuclear-associated membrane by low-speed centrifugations (17,000×*g* for 30 min), the plasma membrane enriched fraction was recovered into the lysis buffer and its protein content was measured with the BCA method (Thermo Scientific Pierce). 40 µg of protein was then denatured, resolved by SDS-PAGE (10% polyacrylamide) and transferred onto a nitrocellulose membrane. Ponceau red labeling was carried out to check for protein loading accuracy. Western blots were performed according to the standard procedure using a polyclonal rabbit anti-ENaC β (StressMarq, Biosciences). For quantification, the band intensities were determined (ImageJ software) and normalized by Ponceau S red intensity.

### Localization of ATP12A on colon slices

Anesthetized mice (10 mg/kg xylazine and 100 mg/kg ketamine) were first perfused with PBS and after with 4% paraformaldehyde (PFA) in the heart to clean and fix tissues. The kidneys were removed and incubated in 4% PFA overnight at 4 °C and then frozen in optimal cutting temperature compound (VWR). 5 µm thick slices were then processed for immunofluorescence microscopy using a homemade anti-ATP12A antibody (1/400^27,65^). For all slides, the pictures were taken with the same parameters of exposition and magnification (× 20). Paraffin-embedded human normal colon slices were purchased at Abcam (ab4327), deparaffinized, rehydrated and processed for immunolabelling with anti-ATP12A antibody (1/400^27,65^).

### Statistical analysis

Results are shown as mean ± s.e.m. Data were tested for significance using two-way ANOVA test followed by a Sidak’s multiple comparisons post-test or Student’s test where appropriate (Prism Software). Outliers were tested by ROUT analysis (Prism Software). The number of mice is indicated in each figure in italic.

## Supplementary information

Supplementary Information
