# Supplementary material for: Increased colonic K+ excretion through inhibition of the H,K-ATPase type 2 helps reduce plasma K+ level in a murine model of nephronic reduction
Source: Sci Rep. 2021 Jan 19;11:1833. doi: 10.1038/s41598-021-81388-0 (PMC7815745; doi:10.1038/s41598-021-81388-0)

Increased colonic K^+ excretion through inhibition of the H,K-ATPase type 2 helps reduce plasma K^+ level in a murine model of nephronic reduction

Christine Walter[1,2], Chloé Rafael[1,2], Anthony Genna[1,2$], Stéphanie Baron[1,2,3] and Gilles Crambert[1,2#]

Supplementary information: Uncropped Western Blots and red Ponceau labelling

A/ Original Western blots and red Ponceau labelling display on Figure 1B.

Two exposures were done on the same film, the one framed in black was displayed in Fig 1B. The first lane, framed in blue, was not related to the Fig 1B.

B/ Original Western blots and red Ponceau labelling display on Figure 4E.

The two gels (a, left part of the Fig 4E, WT mice and b, the right part, HKA2KO mice) were run and transferred in parallel. After red Ponceau labelling, the membranes were physically cut above 50 kDa (see the dash lines) and the upper part was immunolabelled with anti-b ENaC antibody whereas the lower parts were used for other purposes. The black framed parts are the one display in Figure 4E whereas the blue framed parts correspond to another group, not related to the present study.

A Original WB Fig 1B

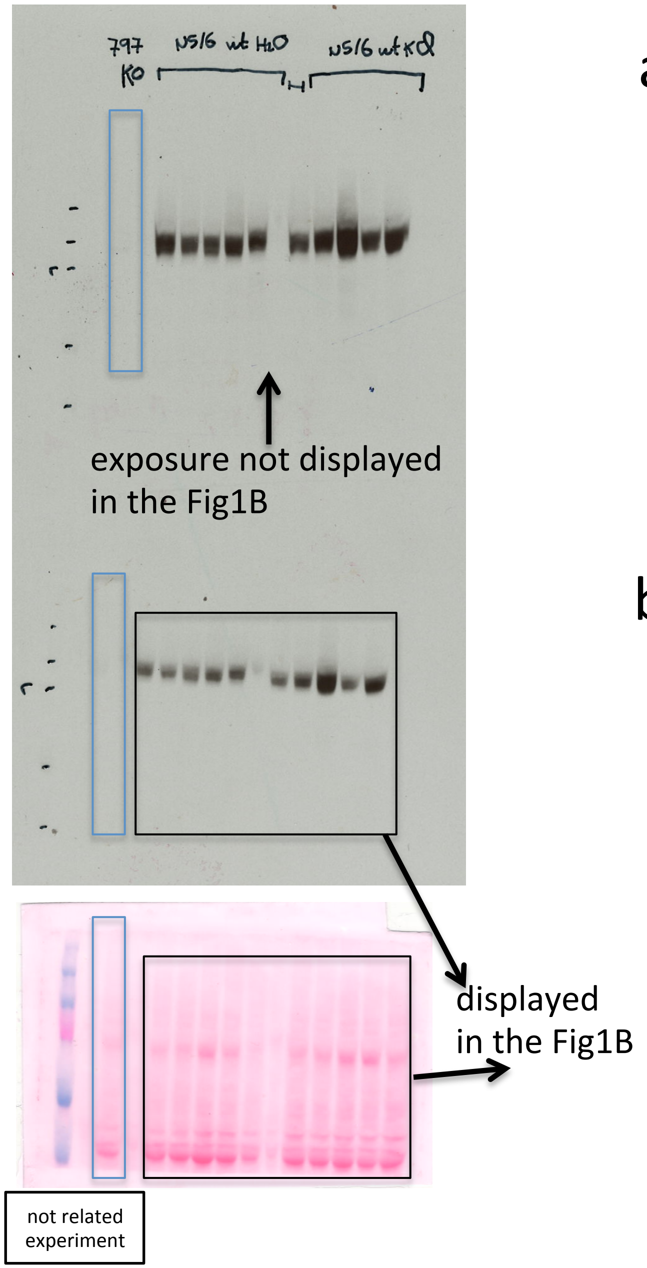

B uncropped WB Fig 4E

a

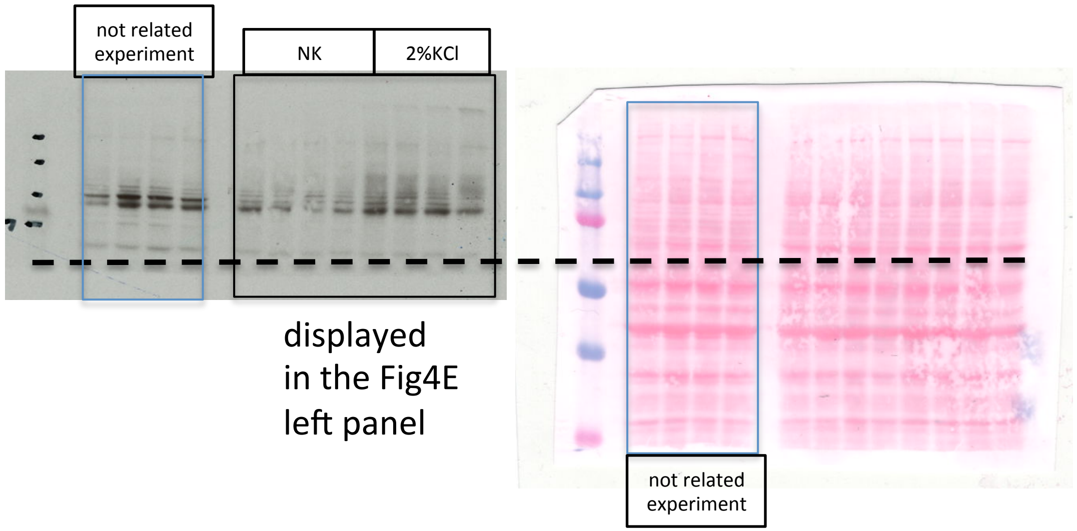

b

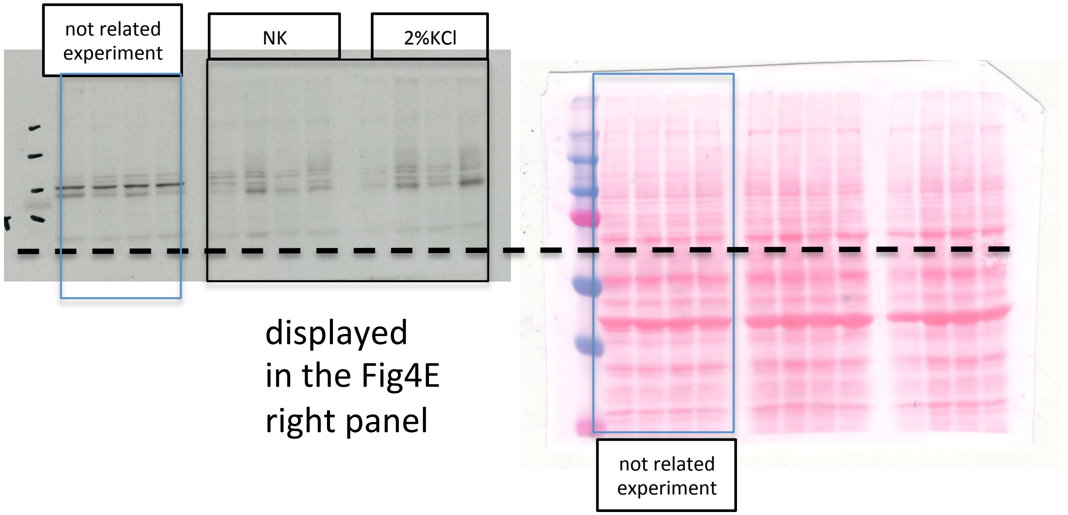

Supplement: Supplementary file 1 — Supplementary Information [file 41598_2021_81388_MOESM1_ESM.pdf]
